# Diffuse Alveolar Hemorrhage As the Presenting Feature of Perinuclear Antineutrophil Cytoplasmic Antibody (p-ANCA)-Associated Vasculitis Preceded by Hearing Loss: A Case Report

**DOI:** 10.7759/cureus.111346

**Published:** 2026-06-23

**Authors:** Khalid F Alhejaili, Khalid G Alabeeri, Basel M Alhejaili, Mohammed N Almohammadi

**Affiliations:** 1 Department of Internal Medicine, King Fahad Hospital, Madinah, SAU; 2 Department of Pulmonology, King Fahad Hospital, Madinah, SAU

**Keywords:** antineutrophil cytoplasmic antibody (anca)-associated vasculitis (aav), bilateral hearing loss, diffuse alveolar hemorrhage, mpo-anca, pulse steroids

## Abstract

Diffuse alveolar hemorrhage (DAH) is a life-threatening pulmonary emergency that may occur as a manifestation of antineutrophil cytoplasmic antibody (ANCA)-associated vasculitis (AAV). We report a rare case of perinuclear antineutrophil cytoplasmic antibody (p-ANCA)-positive vasculitis presenting with severe DAH without renal involvement, preceded by acute bilateral hearing loss. A previously healthy 21-year-old woman presented with hemoptysis, severe hypoxemia, and respiratory failure requiring mechanical ventilation. Chest imaging showed bilateral diffuse infiltrates and ground-glass opacities. Bronchoalveolar lavage findings were consistent with DAH. Serologic workup revealed positive p-ANCA with negative cytoplasmic antineutrophil cytoplasmic antibody (c-ANCA) and anti-glomerular basement membrane (anti-GBM) antibodies. Renal function and urinalysis remained normal throughout admission. The patient was treated with pulse intravenous methylprednisolone followed by oral corticosteroids, leading to rapid clinical improvement and successful extubation within five days. Further history revealed bilateral hearing loss one week before presentation, which may represent a possible early otologic manifestation of vasculitis.

This case highlights that ANCA-associated vasculitis may present as isolated DAH without renal involvement, while unexplained hearing loss may provide an early clue to underlying AAV before the onset of life-threatening pulmonary manifestations. Early recognition and prompt immunosuppressive therapy are essential to improve outcomes.

## Introduction

Diffuse alveolar hemorrhage (DAH) is a fulminant pulmonary syndrome caused by disruption of the alveolar microvasculature and is associated with high morbidity and mortality when recognition is delayed [1,2]. It commonly presents with hemoptysis, hypoxemic respiratory failure, diffuse alveolar infiltrates, and a rapid decline in respiratory status, often requiring intensive care support [1,3]. Among the important etiologies of DAH, antineutrophil cytoplasmic antibody (ANCA)-associated vasculitis (AAV) remains one of the most critical to identify early because timely immunosuppressive therapy can be lifesaving [4,5]. According to the 2012 Chapel Hill Consensus Conference nomenclature, AAV is an umbrella term that primarily comprises small-vessel necrotizing vasculitides associated with ANCA positivity, including microscopic polyangiitis (MPA), granulomatosis with polyangiitis (GPA), and eosinophilic granulomatosis with polyangiitis (EGPA) [4]. Although AAV predominantly affects small vessels, medium-sized arteries may also be involved in some cases [4]. MPA and other ANCA-associated vasculitic disorders typically raise concern when pulmonary hemorrhage occurs in parallel with glomerulonephritis or other systemic manifestations [6]. However, DAH may occasionally emerge as the dominant or initial presentation, creating substantial diagnostic difficulty, particularly when renal function is preserved and extrapulmonary clues are subtle [7,8]. In such settings, the diagnosis may be overlooked initially, especially when the presentation mimics more common causes of bilateral airspace disease [1,2]. We report a young woman who presented with severe DAH requiring mechanical ventilation and was subsequently found to have p-ANCA-positive vasculitis without overt renal involvement. Notably, her illness was preceded by acute hearing loss shortly before the onset of pulmonary hemorrhage, an atypical feature that may represent an early systemic clue to underlying vasculitis. This case highlights the need to consider AAV in any patient with unexplained DAH, even in the absence of renal impairment, and suggests that acute bilateral hearing loss may serve as an early clinical clue to underlying systemic vasculitis before the development of life-threatening pulmonary manifestations.

## Case presentation

A 21-year-old previously healthy, non-smoking woman with no significant past medical history and no regular medication use presented to the hospital with sudden-onset shortness of breath associated with hemoptysis. On initial assessment, she was severely hypoxemic, with an oxygen saturation of 74% on ambient air. Oxygen supplementation was initiated with 5 L/min via nasal cannula; however, persistent hypoxemia required escalation to a non-rebreather mask at 15 L/min.

Vital signs on presentation revealed a blood pressure of 118/72 mmHg, heart rate of 116 beats/minute, respiratory rate of 31 breaths/minute, and temperature of 37.4°C. Physical examination showed a pale and distressed patient with increased work of breathing and use of accessory respiratory muscles. Chest examination demonstrated symmetrical expansion with diffuse bilateral crackles throughout both lung fields. Cardiovascular examination revealed tachycardia with a regular rhythm and normal heart sounds. No peripheral edema, clubbing, rash, purpura, or other skin lesions were observed. Abdominal examination was unremarkable.

Initial laboratory investigations demonstrated a white blood cell count of 12.5 × 10^9^/L, hemoglobin level of 9.4 g/dL, and platelet count of 310 × 10^9^/L. Serum creatinine was 62 µmol/L (reference range: 45-115 µmol/L), while urinalysis showed no evidence of hematuria or proteinuria. Laboratory values are summarized in Table 1.

**Table 1:** Laboratory profile of the patient on admission AST: aspartate aminotransferase; ALT: alanine aminotransferase; PT: prothrombin time; aPTT: activated partial thromboplastin time; INR: international normalized ratio

Laboratory test	Patient value	Reference range
Hemoglobin (gm/dL)	9.4	12-16
Total leukocyte count (cells/mm^3^)	12,500	4000-10,000
Platelet count (cells/mm^3^)	310,000	150,000-410,000
Serum creatinine (µmol/L)	62	53-106
Urea (mmol/L)	3	2.5-6.3
Sodium (mmol/L)	138	136-146
Potassium	4.5	3.5-5.1
AST (U/L)	26	15-37
ALT (U/L)	37	15-60
PT (seconds)	11.9	11.5-15.3
aPTT (seconds)	31	26-40
INR	0.9	0.8-1.2

Initial chest radiography demonstrated diffuse bilateral pulmonary infiltrates (Figure 1).

**Figure 1:**
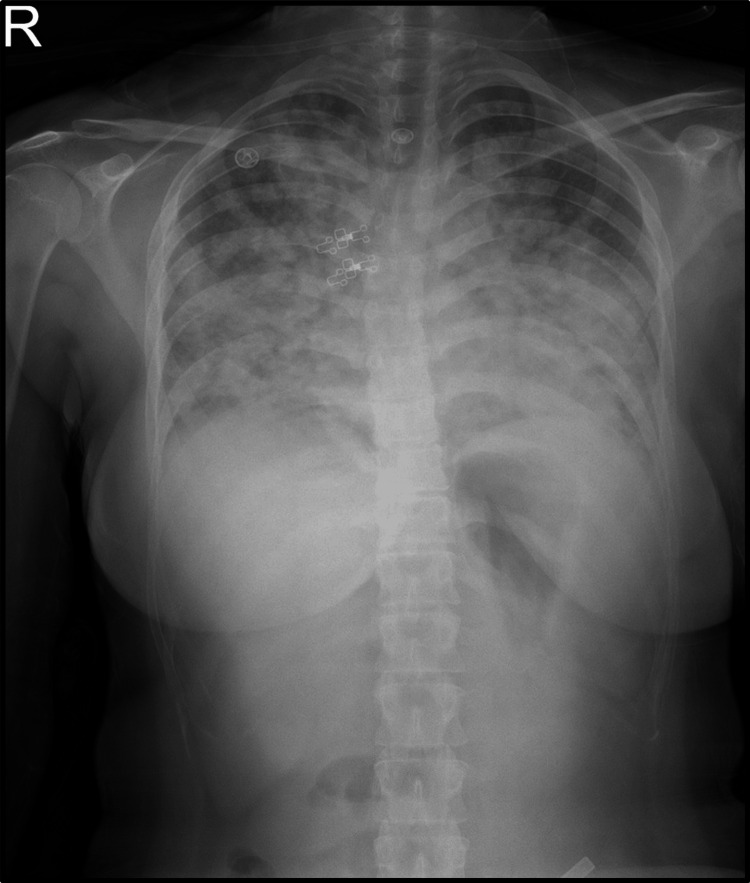
Chest radiograph obtained at presentation demonstrating diffuse bilateral airspace infiltrates consistent with severe alveolar involvement

Chest computed tomography showed bilateral patchy consolidations and bilateral ground-glass opacities (Figure 2).

**Figure 2:**
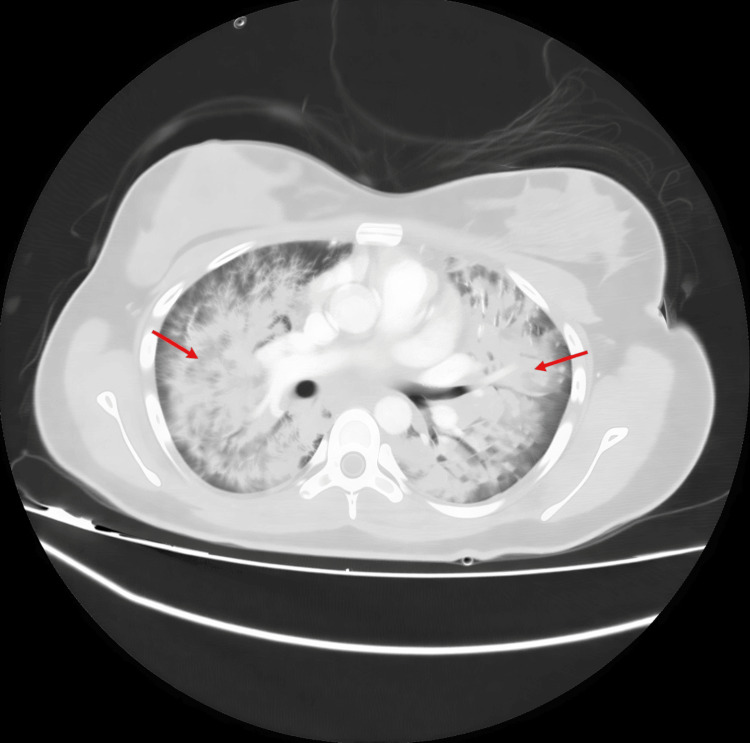
Contrast-enhanced computed tomography of the chest demonstrating extensive bilateral patchy ground-glass and alveolar opacities (red arrows), highly suggestive of diffuse alveolar hemorrhage

Due to progressive hypoxemic respiratory failure, the patient was admitted to the intensive care unit and required mechanical ventilation. A multidisciplinary team consisting of pulmonologists, rheumatologists, and intensive care physicians decided to perform bronchoscopy with bronchoalveolar lavage (BAL) to confirm DAH prior to initiation of pulse corticosteroid therapy. Bronchoscopy performed on the first day of hospitalization demonstrated blood clots within the trachea, limiting complete visualization; however, no focal endobronchial source of active bleeding was identified. Overall findings were consistent with DAH. Given the potential risks associated with immunosuppressive therapy, infectious etiologies were considered and investigated as part of the diagnostic evaluation before initiation of pulse corticosteroid treatment. On the second hospital day, pulse intravenous methylprednisolone therapy was initiated at a dose of 1 g daily for three consecutive days, followed by transition to oral corticosteroid therapy. The patient demonstrated rapid clinical improvement and was successfully extubated on hospital day 5, after which she was transferred to the general ward for completion of further evaluation and management.

Follow-up chest radiography obtained on hospital day 6 showed marked improvement with near-complete resolution of the previously identified bilateral pulmonary infiltrates (Figure 3).

**Figure 3:**
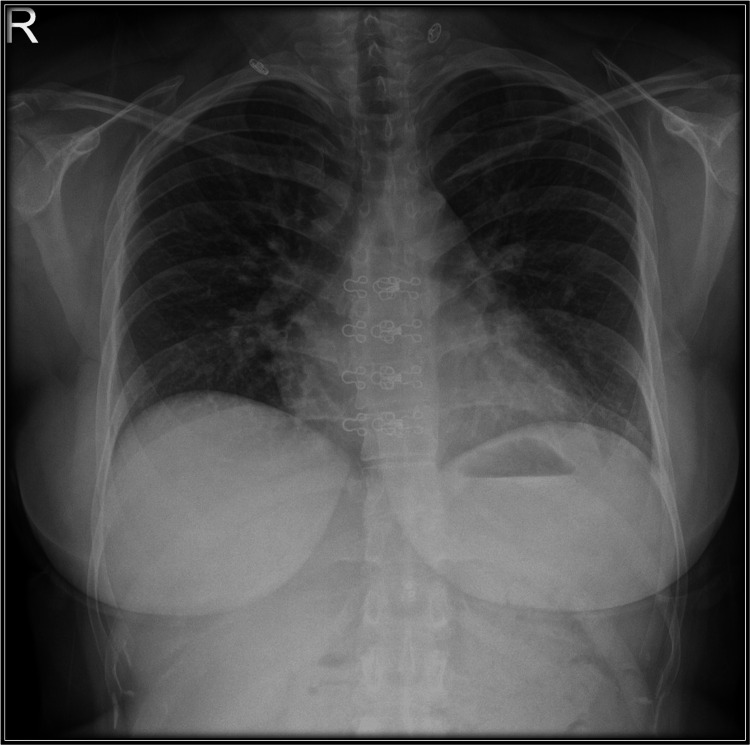
Chest X-ray obtained after pulse corticosteroid therapy demonstrating marked interval improvement with near-complete resolution of the previously identified bilateral pulmonary infiltrates

Further evaluation demonstrated a urine protein-to-creatinine ratio of 0.14 mg protein/mg creatinine (normal <0.2 mg/mg), urine protein of 9.4 mg/dL (reference range: 0-11.9 mg/dL), and urine creatinine of 5985 mg/L (reference range: 2470-19200 mg/L). The infectious workup was unrevealing, with negative tuberculosis PCR testing, no growth in blood cultures, and negative hepatitis serologies. Inflammatory markers revealed an ESR of 14 mm/hour (reference range: 0-20 mm/hour) and an elevated CRP level of 19 mg/L (reference range: 0-5 mg/L).

Further serologic evaluation demonstrated a positive myeloperoxidase (MPO)-ANCA level of 81.4 RU/mL and a negative PR3-ANCA level of 1.88 RU/mL, supporting the diagnosis of MPO-AAV. Anti-glomerular basement membrane antibodies were negative. Following confirmation of AAV, induction therapy with rituximab was initiated at a dose of 1 g intravenously, followed by a second 1 g intravenous dose 14 days later. Maintenance therapy consisted of rituximab 500 mg every six months. In addition, avacopan 30 mg orally twice daily was prescribed as a steroid-sparing agent. Oral corticosteroids were gradually tapered and successfully discontinued after four months. Renal function remained preserved throughout hospitalization. After clinical stabilization, a more detailed history revealed that the patient had experienced acute bilateral hearing loss approximately one week prior to presentation. Prior to her current presentation, the patient underwent otolaryngologic evaluation for chronic bilateral tinnitus and hearing impairment. Audiometric testing demonstrated mild-to-moderate low-frequency conductive hearing loss in the right ear and mild-to-moderate mixed hearing loss in the left ear, with normal tympanometry bilaterally.

The patient was subsequently weaned off supplemental oxygen and was planned for discharge with close outpatient follow-up. Upon discharge, the patient was maintained on avacopan 30 mg orally twice daily and rituximab 500 mg administered every six months for remission maintenance. Oral corticosteroid therapy was continued on a gradual tapering schedule, beginning with prednisone 60 mg daily, with progressive dose reduction until complete discontinuation after four months. During six months of follow-up, the patient remained in clinical remission without evidence of vasculitis flare, recurrent DAH, or new organ involvement. Subsequent follow-up by the ENT service demonstrated persistent symptoms without significant clinical improvement.

## Discussion

DAH is a rare but potentially catastrophic pulmonary syndrome caused by disruption of the alveolar-capillary basement membrane, leading to blood accumulation within the alveolar spaces [1]. Clinically, DAH commonly presents with hemoptysis, progressive dyspnea, hypoxemic respiratory failure, diffuse pulmonary infiltrates, and acute anemia [1,3]. Because these manifestations overlap with more common pulmonary disorders such as severe pneumonia, pulmonary edema, and acute respiratory distress syndrome, diagnosis is often delayed, which contributes to its high morbidity and mortality [3]. Careful exclusion of infectious causes is particularly important before initiation of high-dose immunosuppressive therapy, as unrecognized infection may lead to clinical deterioration and potentially fatal outcomes [1,3]. Prompt recognition is therefore essential, particularly when respiratory failure develops rapidly [1,3]. Among immune-mediated causes, AAV remains one of the most important etiologies to identify early because immunosuppressive therapy can be lifesaving [5,8].

MPA, a pauci-immune necrotizing small-vessel vasculitis commonly associated with MPO/p-ANCA positivity, classically involves the kidneys and lungs [6,9]. Renal involvement, typically manifesting as rapidly progressive crescentic glomerulonephritis, is considered a hallmark feature and is frequently accompanied by pulmonary capillaritis causing DAH [6,9]. However, our case illustrates an atypical presentation in which fulminant pulmonary hemorrhage occurred in the absence of overt renal involvement, with preserved renal function and no hematuria or proteinuria at presentation. This renal-sparing phenotype is uncommon and presents a diagnostic challenge because clinicians often rely on concomitant pulmonary-renal syndrome as a clue toward vasculitis [10]. The absence of renal manifestations in our patient could easily have delayed consideration of AAV as the underlying cause.

A particularly notable feature in our case was the history of acute bilateral hearing loss one week prior to presentation. Otologic manifestations, including sensorineural hearing loss, otitis media, and vestibular dysfunction, are well recognized in AAV, particularly in GPA, but are less commonly emphasized in p-ANCA/MPO-positive vasculitis [11]. Auditory symptoms may reflect small-vessel inflammation involving the cochlear microcirculation or vasculitic involvement of adjacent structures [11]. In retrospect, this localized symptom may have represented an early systemic manifestation of vasculitis preceding the development of life-threatening pulmonary capillaritis. Recognition of such subtle extrapulmonary clues may facilitate earlier diagnosis in atypical presentations.

Compared with the previously reported case by Behera et al., which described unilateral DAH as the presenting feature of p-ANCA-positive MPA with severe renal dysfunction requiring plasmapheresis, cyclophosphamide, and pulse corticosteroids [12], our patient demonstrated a distinctly different phenotype. Although both patients shared p-ANCA positivity and DAH as the dominant presentation, our case involved bilateral DAH with preserved renal function, highlighting the heterogeneity of organ involvement in MPO-positive vasculitis [10,12]. Furthermore, our patient showed rapid and marked clinical improvement with pulse methylprednisolone therapy alone, achieving successful extubation within five days. This favorable response suggests that early recognition and immediate immunosuppression may rapidly reverse pulmonary inflammation when irreversible end-organ injury, particularly renal damage, has not yet developed [5,9].

This case emphasizes several important clinical pearls. First, AAV should remain in the differential diagnosis of any unexplained DAH, even in young patients or in the absence of renal impairment [9,10]. Second, localized symptoms such as sudden hearing loss may represent early vasculitic manifestations and should not be overlooked [11]. Finally, multidisciplinary collaboration among intensivists, pulmonologists, and rheumatologists may facilitate earlier diagnosis and timely initiation of therapy in potentially life-threatening presentations such as DAH associated with AAV [1,5].

Limitations of this report include its reliance on a single patient, which limits the ability to generalize these findings to the broader population of patients with AAV. An additional limitation is the lack of detailed audiologic documentation, including formal audiometric findings, which limits complete characterization of the reported hearing loss and assessment of its relationship to the underlying vasculitic process. Although the patient was evaluated by the otolaryngology (ENT) service, comprehensive audiologic data were unavailable for review. Further studies are needed to better characterize the spectrum of otologic manifestations in AAV and their potential role in disease presentation and recognition.

## Conclusions

DAH is a life-threatening pulmonary emergency that requires prompt recognition and treatment. Early diagnosis and timely initiation of immunosuppressive therapy may significantly improve clinical outcomes and survival. AAV should remain an important differential diagnosis in patients presenting with unexplained DAH, even in the absence of renal involvement. In addition, unexplained hearing loss should prompt consideration of systemic vasculitis in the appropriate clinical context, particularly when accompanied by systemic symptoms.
